# Interventions with Social Integration Components Addressing Psychosocial Outcomes of Young- and Middle-Aged Adult Cancer Individuals: A Systematic Review

**DOI:** 10.3390/cancers15194710

**Published:** 2023-09-25

**Authors:** Pragya G. Poudel, Madeline R. Horan, Tara M. Brinkman, Zhaoming Wang, Leslie L. Robison, Melissa M. Hudson, I-Chan Huang

**Affiliations:** 1 Department of Epidemiology and Cancer Control, St. Jude Children’s Research Hospital, 262 Danny Thomas Place, Memphis, TN 38105, USA; pragya.gautampoudel@stjude.org (P.G.P.); madeline.horan@stjude.org (M.R.H.); tara.brinkman@stjude.org (T.M.B.); zhaoming.wang@stjude.org (Z.W.); les.robison@stjude.org (L.L.R.); melissa.hudson@stjude.org (M.M.H.); 2 Department of Psychology and Biobehavioral Sciences, St. Jude Children’s Research Hospital, Memphis, TN 38105, USA; 3 Department of Computational Biology, St. Jude Children’s Research Hospital, Memphis, TN 38105, USA; 4 Department of Oncology, St. Jude Children’s Research Hospital, Memphis, TN 38105, USA

**Keywords:** cancer, systematic review, social integration, social connectedness, interventions

## Abstract

**Simple Summary:**

Adult cancer patients and survivors often experience social integration and connectedness chal-lenges. This systematic review summarizes the effect of social integration or social connectedness interventions among young- and middle-aged cancer patients and survivors based on 28 empirical studies published between 2000 and 2021. We found that social integration interventions that utilize technology- and/or non-technology-based platforms show improved social outcomes, increased awareness about available cancer-related resources, decreased perceived isolation, increased knowledge and access to cancer survivorship resources, and improved patient-reported outcomes among cancer individuals versus the comparison individuals. We recommend utilizing suitable platforms, whether technological or non-technological, to facilitate connections between cancer patients/survivors with friends, fellow cancer patients, or society members. This will enable pa-tients/survivors to access essential resources and support, thus enhancing their ability to cope with challenging life situations and ultimately improving social well-being and health outcomes.

**Abstract:**

Background: The majority of adult cancer patients/survivors encounter social challenges (e.g., obtaining social support, maintaining social relationships, feelings of social isolation). This systematic review summarizes intervention studies addressing social integration or social connectedness issues among young- and middle-aged cancer patients/survivors. Methods: We searched the PubMed, CINAHL, and Web of Science databases (January 2000–May 2021) to identify intervention studies that addressed social integration, social connectedness, social support, and social isolation for cancer patients/survivors in young- and middle-aged adulthood (18–64.9 years) through a randomized controlled trial (RCT). We categorized the interventions as technology-based, non-technology-based, and mixed-type (technology- and non-technology-based). Results: A total of 28 studies were identified. These interventions demonstrated improved social outcomes (e.g., increased social support, decreased loneliness), increased awareness of available cancer-related resources, and better patient-reported outcomes among patients/survivors versus controls. Specifically, the use of internet-based discussion sessions was associated with improved social cohesion and social support. Receiving social support from peers through networking sites was associated with improved physical activity. Additionally, implementing mixed-type interventions led to better social support from peer survivors, less fear of social interactions, and improved social connectedness. Conclusions: Using existing technology- and/or non-technology-based platforms to facilitate social connectedness among cancer patients/survivors in young- or middle-aged adulthood can help them cope with stressful life circumstances and improve quality-of-life. Further interventions targeting social integration (e.g., social network interventions) are needed to improve the complex social integration challenges experienced by cancer patients and survivors.

## 1. Introduction

Cancer is the second leading cause of death in the United States [1]. With improvements in treatments, the 5-year survival rate is increasing [2]. However, adverse treatment effects during therapy and late effects that occur during survivorship often impact social relationships with partners, family members, and friends. A study of people with adult-onset cancer found that almost 50% of patients experienced social difficulties, including problems obtaining social support, maintaining social connectedness, and perceptions of social isolation and restrictions in work and social activities [3]. Poor social functioning is associated with impaired quality-of-life, especially among ethnic minorities in cancer populations [4]. Psychosocial intervention (e.g., providing cancer information, cognitive–behavioral skills training, psychotherapy) is one strategy for improving social functioning and quality-of-life among cancer patients and survivors [5]. Many psychosocial interventions for cancer patients and survivors are based on social cognitive theory, which posits that improved outcomes occur in the social context and are driven by self-efficacy, outcome expectations, and self-regulation [6,7,8]. 

Maintaining healthy social networks facilitates positive lifestyle changes and optimal physical and psychological well-being [9]. A meta-analytic study found that the survival rate was 50% greater among cancer patients who had strong social ties compared to those with weak ties [10]. Individuals with optimal social relationships typically receive appropriate social resources (e.g., financial assistance), informational/emotional support (e.g., advice-giving, empathy), and instrumental support (e.g., help with daily chores) that further improve neuroendocrine responses and buffer acute or chronic stressors [10,11]. Cancer patients and survivors may receive increased support and care from others immediately after the diagnosis of cancer; however, these supports often decline gradually, and this shift in support can lead to increased feelings of loneliness over time [12]. In evaluating the mechanistic influence of childhood cancer experiences on subsequent patient-reported outcomes (PROs), a recent study found that greater personal social connectedness can potentially buffer the negative effects of the cancer experience on poor physical and mental functioning [13]. This finding suggests the need for interventions that include strategies to address the unique social integration challenges reported by individuals with cancer. The strategies used in psychosocial interventions based on social cognitive theory for this population include improving social support and coping skills, access to appropriate resources, role-playing, and testimony from cancer survivors and patients [6]. Using a theoretical framework (e.g., social cognitive theory, experiential–existential theory) as the blueprint for an intervention is helpful in guiding the selection of intervention methods to improve the intended outcomes [6,7,14].

Young- and middle-aged cancer patients and survivors (18 to 64.9 years of age) experience unique social challenges [15]. The social development of this age group is characterized by intimacy (i.e., seeking enduring relationships with friends, family members, and coworkers) and work-related accomplishments (i.e., having a meaningful job) [16]. Young- and middle-aged cancer patients and survivors may experience disruptions in developing intimate and productive social relationships, as they might find difficulties in navigating complex social situations due to poor physical and mental health conditions (e.g., cognitive late effects, body image issues, anxiety/depression, fear of cancer recurrence) from cancer and associated treatments. These health conditions may also lead to difficulties in achieving independence from family or the inability to gain employment [17]. These issues can be addressed through interventions aimed at improving social relationships with friends, family members, and coworkers, which is a strategy in social cognitive theory-based interventions [17]. People of this age range have high digital literacy and use technology and social media daily; therefore, technology-based interventions may be an effective avenue for improving social connection. The literature on whether technology alleviates or reinforces loneliness is mixed in adults [18,19], but evidence shows that specifically in young adults, technology can improve social connection [20].

Several systematic review studies have reported the effects of social interventions on health outcomes for patients and survivors with specific cancer diagnoses (e.g., prostate [21,22] or gynecological [23] cancer). However, these interventions focused on older patients/survivors. Additionally, previous review studies did not address different effects of interventions by the type of approaches or platforms used in the intervention (e.g., technology-based, non-technology-based communication). This systematic review aimed to identify studies describing randomized controlled trials (RCTs) that evaluated the effects of interventions with social integration or connectedness components and summarize the social and health-related endpoints of these interventions among cancer patients and survivors in young- or middle-aged adulthood. We hypothesized that cancer patients and survivors who participated in social integration or connectedness interventions would be more likely to experience better social and health-related outcomes compared to the participants in control groups. Additionally, we hypothesized that the social integration or connectedness interventions delivered in person, using a non-technology platform, would provide better social connection among cancer patients and survivors, resulting in better social and health-related outcomes as compared to those who used a technology platform.

## 2. Methods

We searched PubMed, CINAHL, and Web of Science between 1/1/2000 and 5/31/2021 to identify studies that met the following inclusion and exclusion criteria (see Supplemental Table S1 for the search terms). Articles included in this study were original studies: (1) addressing social integration, social connectedness, social support, or social isolation problems as either the primary or secondary outcome among cancer patients and/or survivors who were in young- and/or middle-aged adulthood (mean age 18 to 49.9 years in each study to ensure the age range of all study participants did not extend past middle-age, which ends at 64.9 years) [15]; (2) incorporating at least one social intervention component based in social cognitive theory identified through a previous meta-analysis [6] (i.e., relaxation training; physical, affect, or spiritual coping; practice new skills; role-playing; modeling of behavior; cognitive restructuring; self-monitoring of skills/thoughts; goal setting; setting realistic expectations; cancer survivor or patient testimony; self-help materials; problem solving); (3) describing interventions that used an RCT design that randomly assigned participants into an experimental or control group; and (4) available in full text and published in English. Articles excluded from this study were those: (1) reporting interventions without a social interaction component (e.g., no group-based activities, only unidirectional communication); (2) reporting interventions focused only on family members or caregivers; (3) limited to observational outcomes; and (4) being non-empirical/non-full-length (e.g., conference paper/proceeding, abstract only, letter to the editor, commentary, protocol). The Preferred Reporting Items for Systematic Reviews and Meta-Analyses criteria were used to guide the systematic review [24].

Studies were chosen for inclusion by the first and senior authors, and any disagreements were resolved by discussion. In step 1, the literature searches described above yielded 1483 studies. In step 2, 79 duplicates were removed, leaving 1404 studies for further assessment. In step 3, titles and abstracts were screened, and 34 studies meeting the inclusion/exclusion criteria were retained. In step 4, full-text studies were assessed, and 28 articles from 25 different RCTs were included in this systematic review (Supplemental Figure S1).

Data extracted from original studies included authors, publication year, country of publication, study aim, demographic characteristics of participants, cancer diagnosis, intervention design, intervention platform, the intervention component based on social cognitive theory, outcomes assessed in the intervention, main results, and implications. In this review, the endpoint of our primary interest was social outcomes (e.g., social connectedness, integration, functioning). In addition, we reviewed other endpoints (e.g., lifestyle/health behavior, PROs, survival status) for the interventions that had a social integration component but did not assess social outcomes as the primary endpoint.

The quality of included studies was assessed using the Cochrane Risk of Bias tool [25]. This tool is used to examine seven categories related to the design, conduct, analysis, and presentation of factors that might cause the effect of an intervention to be over- or underestimated. For each study, each of the seven categories was rated as low, high, or unclear based on the levels of bias.

## 3. Results

Among the 28 studies included in this systematic review (Table 1), the quality assessment suggested a low likelihood of bias in our review (Supplemental Table S2). Of the included studies, twelve were conducted in the USA [26,27,28,29,30,31,32,33,34,35,36,37]; three in China [38,39,40]; two in Canada [41,42], Iran [43,44], the Netherlands [45,46], and South Korea [47,48]; and one in the UK [49], Australia [50], Germany [51], Hong Kong [52], and Taiwan [53]. The duration of interventions ranged from 4 weeks [28] to 26 weeks [29]. The mean (±SD) ages of the participants included in the interventions ranged from 21 (±5) [27] to 50 (±11) years [36]. Control groups received standard care educational materials, usual routine care, or were on a wait-list for the intervention. In studies where control group participants were on a wait-list, several studies mentioned that members of the control group were offered the option to participate in the intervention program after the completion of the study [28,36,39,41,44,45,51].

Fifteen studies used various psychological and behavioral theories to facilitate the design of their interventions. Specifically, four studies [30,31,32,48] relied on social cognitive theory to design an intervention for improving survivors’ learning in social contexts. Two studies were based on a cognitive–behavioral, problem-focused model [26,36]. Two studies described an intervention based on an experiential–existential theory with cognitive–behavioral components [45,46]. Three studies used self-efficacy theory to design the intervention [28,42,47]. One study used a psychophysiology framework to examine the psychological and physical effects of psychosocial intervention [52]. One study adopted social interaction theory [53] to improve survivors’ health behaviors and social integration through engagement and interactions with other survivors. One study [38] adopted the health belief model to improve patients’ health behaviors and outcomes. One intervention was guided by the resilience model for individuals with breast cancer [40].

The platform used for implementing interventions was classified into three categories: 11 technology-based [26,27,28,29,30,31,32,33,38,41,42]; 13 non-technology, face-to-face-based [34,35,36,37,39,43,44,45,46,49,50,51,52]; and 4 mixed-type (technology-based and non-technology-based) interventions [40,47,48,53]. Of the technology-based interventions, nine used the Internet, online chat/discussion, or Facebook platforms [28,29,30,31,32,33,38,41,42], and two used telephone communication [26,27]. The technology-based interventions were published between 2001 and 2020, the studies describing non-technology-based interventions were between 2004 and 2020, and the mixed interventions were between 2012 and 2019. For the mean duration of interventions, technology-based interventions lasted for 13.5 weeks, non-technology-based interventions for 10.8 weeks, and mixed interventions for 9.5 weeks.

Table 2, Table 3 and Table 4 summarize the effects of the technology-based, non-technology-based, and mixed-type interventions, respectively. Regarding the endpoints of the studies, 13 studies reported social outcomes. Studies also reported results of other endpoints, including PROs (*n* = 26), lifestyle (*n* = 4), health-related resources (*n* = 1), and survival status (*n* = 2).

### 3.1. Technology-Based Interventions (Table 2)

#### 3.1.1. Internet, Online Chat/Discussion, or Facebook Groups

Four studies described the effects of technology-based interventions on social outcomes [28,29,32,42]. Using a culturally tailored, internet-based cancer support program for pain management among Asian American cancer survivors, one study showed no significant differences in pain control, although there was a significant decrease in perceived isolation and the degree of uncertainty and an increase in personal resources in the intervention group versus the control group [28]. Another study found that sharing personal stories, providing local resources (e.g., cancer-related news, articles, videos), and offering coping advice (e.g., sharing experiences, building self-efficacy) resulted in better peer social support and information literacy in the intervention group versus the control group [29]. In another study, adolescent and young adult (AYA) survivors who used an online platform featuring a group chat and emotion-focused group discussion videos had significantly higher social cohesiveness compared to survivors who were provided a platform with the group chat and an educational workbook alone [42].

Three studies focused on the effects of an intervention on physical activity outcomes in AYA cancer survivors [30,31,32]. This intervention adopted Facebook group-based interventions, which connected peers through online posts and resource sharing (e.g., cancer-related news articles, videos, physical activity resources). The efficacy of survivors’ engagement in a networking intervention group (i.e., study coordinators-initiated discussions) versus a self-help control group (i.e., self-initiated discussions or interactions by participants) showed no statistically significant differences in weekly moderate-to-vigorous physical activity in both groups. However, the participants in the intervention group had greater weight loss over time versus participants in the control group [31]. Another study found that the dissemination of group posts initiated by cancer survivors, rather than by the moderator, tended to increase group engagement and interactions, which promoted physical activity [30]. The effects of the intervention on physical activity were mediated by changes in self-efficacy, social support, and self-monitoring of physical activity [32]. Compared to the control group, where participants self-initiated the social interactions, the participants in the intervention group, where the study administrator moderated the interactions, showed lower self-efficacy for adhering to the exercises and receiving social support from Facebook friends.

Six studies reported the effect of interventions on PROs [28,29,31,33,38,41]. In one study, participants in the intervention group, who were encouraged to interact with peers to share emotional distress, treatment experiences, frustrations, fear of recurrence, self/body images, romantic relationships, relationships with friends/family members, and coping strategies, had a significantly lower risk of depression, cancer-related trauma, and perceived stress versus the control group [33]. Online discussion forums and supportive group psychotherapy for sexual concerns provided comfortable environments to discuss sexual issues and mitigate sexual-related psychological distress [41]. Another study that used online discussion forums and group therapy found that the intervention group reported lower cancer-related uncertainty compared to the control group [28]. Similarly, in another study, physical, psychological, and social rehabilitation and sharing coping strategies with peers showed improvements in physical, social, and emotional well-being [38]. An intervention providing informational, social support, and decision-making services led to greater information literacy and confidence in communicating with their doctors [29].

#### 3.1.2. Telephone-Based Interventions

Two studies used telephones to deliver the interventions [26,27]. In one study, interventions conducted over the telephone to promote cancer-related communication skills, coping skills, balancing emotions, and stress management through social support were associated with increased physical well-being and overall quality-of-life [26]. Another study with two intervention arms (i.e., text messaging and peer navigation) found that members who participated in the text messaging intervention group increased survivorship care knowledge versus those in the control group [27]. Survivors in the peer navigation intervention group (i.e., survivors matched with a navigator who used telephones to identify survivorship-related resources in the community) exhibited higher self-efficacy for survivorship care versus the control group. Additionally, participants in both intervention arms had higher motivation towards seeking survivorship care versus the control arm (text messaging group Cohen’s d = 0.70 and peer navigation group Cohen’s d = 0.68; Table 2).

### 3.2. Non-Technology-Based Interventions (Table 3)

In the non-technology-based interventions, six studies focused on the effects of interventions on social outcomes [35,44,45,49,51,52]. Participants in a mindfulness-based stress reduction class had better social well-being compared to the control group [49]. An intervention that incorporated Western psychotherapeutic elements with Eastern health practices (e.g., normalizing traumatic experiences, forgiveness, and self-love as means to peacefulness, reinforcement, and stabilization through social support and helping others) led to better receipt of social support compared to the control group [52]. A group dance class intervention led to better social functioning compared to the control group [51]. A study found that an intervention that encouraged participants to increase their social interactions and build new social bonds decreased loneliness and increased hope compared to the control group [44]. However, in another study, an in-person-based approach to providing social support and coping skills showed no difference in psychosocial adjustment, although participants in the intervention group perceived more social support from others versus the control group [45]. Another in-person-based group therapy intervention (e.g., creating a supportive environment to express experiences, discussing body image challenges, dealing with fears of death, reordering life priorities) also showed no significant differences in social support unless an extreme outlier was included in the analysis [35].

All 13 non-technology-based studies evaluated the effects of social interventions on PROs [34,35,36,37,39,43,44,45,46,49,50,51,52]. In one study, compared to the control group, the intervention group that used educational sessions to improve communication and coping skills, provided nutritional materials to improve adherence to a healthy diet, and created booklets or brochures to direct cancer patients to local resources showed improvements in physical and mental well-being [34]. In-person-based interventions aimed at building new connections and sharing fears of death showed decreases in anxiety and depression [35,49]. For ethnic minorities, providing culturally and linguistically appropriate emotional support to Spanish-speaking cancer survivors, sharing cancer-related information, and improving emotional support and acceptance of cancer improved emotional well-being in the intervention group versus the control group [36].

In another study, the use of in-person meditation practice plus supportive group discussions regarding cancer experiences and challenges decreased perceived stress among participants in the intervention group (Cohen’s d = 0.38) versus the control group [39]. Group interventions to decrease stress and increase emotional coping were associated with better management of cancer-related stress and coping strategies [43,45]; provision of emotional support and emotional encouragement were associated with better body image, recreational activities, and quality-of-life [44,46]. Peer counseling from other cancer survivors, in conjunction with an informational workbook, led to decreased emotional distress compared to the control group [37]. Participating in a social network for building new social support bonds, improving relationships with friends/family members, enhancing communication with healthcare providers about symptoms, and gathering information and beneficial resources showed an improvement in quality-of-life and a decrease in fatigue and anxiety [49,50,51,52].

### 3.3. Mixed-Type (Technology- and Non-Technology-Based) Interventions (Table 4)

Among the four studies that adopted both technology- and non-technology-based designs in the interventions, two studies focused on PROs and social outcomes [40,53], and two studies focused on PROs only [47,48].

In the studies that reported both PROs and social outcomes, the interventions that were designed to facilitate information-sharing regarding cancer treatment and side effects through in-person meetings and/or phone calls to review the provided materials and provide opportunities for social interactions and communication with others showed less fear and avoidance of social interactions in the intervention group versus the control group [53]. A mentor–mentee matching program that provided both in-person and telephone contact to share personal feelings among participants who may feel uncomfortable sharing in a large group found a reduction in depression (Cohen’s d = 0.65) and an increase in hopefulness two months post intervention (Cohen’s d = 0.81) and a decrease in anxiety (Cohen’s d = 0.74) and fatigue (Cohen’s d = 0.65) and better social support (Cohen’s d = 0.51) and resilience (Cohen’s d = 0.83) six months post intervention, compared with the control group [40].

In the studies focusing on PROs as endpoints, cancer patients supported by peers with empathy and encouraged to share emotions and promote self-confidence increased self-efficacy for disease management versus patients in the control group [47]. In another study, participants in the intervention group, who received handbooks about cancer experiences and engaged in coaching sessions and small group meetings, where they discussed emotional stress, diet, and exercise, had higher quality-of-life and emotional well-being and decreased psychological distress versus the control group [48].

## 4. Discussion

Among 28 intervention studies from 25 RCTs that addressed social integration and connectedness issues in young- and middle-aged adult cancer patients and survivors, we found that the use of technology- and/or non-technology-based platforms improved social outcomes, knowledge of and access to survivorship care resources, and various domains of PROs in individuals with cancer compared with controls. While it would have been informative to compare specific outcomes from technology- vs. non-technology-based interventions, it was not possible in this review because the outcomes were not comprehensively evaluated in both types of platforms.

Affective coping was the most common intervention component based on social cognitive theory included in the technology-based and non-technology-based interventions. Specifically, technology-based interventions included more cancer survivor/patient testimony, whereas non-technology-based interventions included more relaxation training, spiritual coping, and opportunities to practice new skills. However, many studies did not explicitly provide any rationale for the study design or intervention methods, based on social cognitive theory or any other theoretical framework. The technology-based, non-technology-based, and mixed-type interventions all had unique platform design and operation features that made the interventions successful, which are discussed below.

### 4.1. Technology-Based Interventions

The technology-based approach provides opportunities for cancer patients/survivors to interact with each other in ways that improve social connectedness, without constraints on location or time of day. Technology-based interventions have shown decreased loneliness and enhanced certainty about life circumstances and future health status. Interacting with peers and sharing personal stories and coping strategies improved PROs, suggesting that survivor navigators help address the health-related needs of cancer patients and survivors [33].

As technology-based interventions are not bound by time or geographical locations, adopting mHealth or internet-driven interventions could be beneficial, especially for young adult survivors who use advanced technologies in their day-to-day life. In internet-based interventions, participants were encouraged to maintain communication and share resources on websites, although these interventions often did not report whether the posted information and shared resources were appropriate for study participants. The participants were given links to publicly available physical activity and cancer survivorship resources; however, the utilization of these resources was not evaluated. Additionally, prior studies have shown that internet-based interventions have high rates of attrition, especially among participants who might not be comfortable using technology [54,55]. Thus, it is important to consider the participants’ experience with and preference for the technology in the design of the intervention. Related factors that warrant consideration include age, cultural background, technology literacy, and privacy concerns about sharing their experiences online [56].

### 4.2. Non-Technology-Based Interventions

Non-technology-based interventions focus on improving social interactions and building new social bonds through in-person interactions. Participants were often given supportive booklets, brochures, and local resources; however, the utilization of these materials and resources was not evaluated in these studies. Furthermore, evidence of the sustainability and accessibility of these materials and resources post interventions was not described.

Face-to-face interventions were most often facilitated by healthcare professionals as the mechanisms of intervention (e.g., managing cancer-related challenges, coping with problems, improving relationships with friends/family, finding local resources); therefore, participants had the opportunity to discuss their feelings and emotional experiences, and receive helpful resources from professionals. Structured sessions (e.g., professionals presented informational materials, followed by the program facilitator guiding group discussions, case activities, and exercises) were beneficial to patients’ and survivors’ outcomes, and the addition of unstructured open-expression and open-communication sessions (e.g., providing participants the opportunity to share cancer-related experiences and interact with other patients/survivors to form new bonds of social support) also improves long-term social connectedness [33,44]. Participation in emotional expression through discussion sessions with other survivors may lead to new friendships and sources of social support, which eventually promote an individual’s perception of social belonging, psychological well-being, and better quality-of-life [44]. Importantly, conducting culturally and linguistically appropriate interventions by peers and professionals of a similar cultural background can help to inspire, educate, and instill hope through a culturally inclusive and comfortable context [28].

### 4.3. Mixed-Type Interventions

Mixed-type interventions often rely on both face-to-face and telephone-based approaches, providing the opportunity for communication with peers or mentors. Studies that adopted the mixed-type approach demonstrated positive outcomes over time [40,53]. For example, Ye et al. [40] showed no significant difference observed immediately after the intervention, but a significant decrease in depressive symptoms two months post intervention and anxiety and fatigue six months post intervention in the intervention group compared to the control group. This finding is consistent with a previous systematic review on prostate cancer survivors, which showed improved psychosocial outcomes three months after the intervention [21]. Forming new social bonds with others is often a gradual process, so it is important to design longitudinal social integration interventions with longer follow-ups and evaluate the sustainability and long-term effects for cancer populations.

### 4.4. Implementation Challenges of Technology- and Non-Technology-Based Interventions

Although the effects of technology- and/or non-technology-based interventions have shown promising outcomes, it is important to understand the feasibility and implementation challenges related to these methods. Based on the 28 studies included in our review, culturally tailored social interactions through technology-based interventions were feasible and effective in distributing health information and improving health outcomes. Also, group cohesion and connectedness through technology-based platforms were found to be relevant, especially for AYA cancer patients/survivors. Technology-based interventions could be valuable over face-to-face interventions when recruiting participants who reside in geographically remote locations (e.g., rural areas). Importantly, technology-based interventions may be more useful if cancer patients/survivors are physically disabled or required to maintain physical distance (e.g., during the COVID-19 pandemic). However, all intervention participants should be comfortable using the internet, computers, or electronic/mobile devices. To address technological barriers, it is useful to account for the preferences of study participants in the design of electronic devices or platforms before implementing interventions.

Face-to-face-based interventions can be applied to all age groups; however, study participants need to be present at the intervention site at the pre-specified time. Despite these challenges, verbal and non-verbal approaches used to communicate with others during face-to-face encounters play a critical role in building trust, sharing emotional support, and inspiring hopefulness. Some studies have shown that social ties and friendships initiated during face-to-face interventions can last several years post intervention [44]. To increase dynamic interactions among participants, the intervention facilitator can apply strategies or techniques (e.g., asking participants about their cancer experiences or challenges, sharing news or current cancer-related resources, sharing inspirational videos) to encourage participants to engage in group discussions or interactions. Informal coffee or snacks break between intervention sessions can also help increase interactions among participants and build social connections [45,46,52]. In addition, no interventions used a modern technology platform (e.g., Zoom, Skype, FaceTime) to initiate virtual interactions. Future interventions should assess the feasibility of these platforms in delivering social interventions to cancer patients/survivors.

The interventions described in this review study fall under the umbrella of traditional psychosocial interventions, which are most often developed to improve psychosocial outcomes. These outcomes are measured through standard psychosocial or PRO surveys that contain a social domain (e.g., the role-functioning domain of the SF-36), but they are not designed to measure multifaceted, dynamic social connectedness. Interventions that address the complexity of social integration outcomes are needed to fully understand social networks’ role in health outcomes. The social interactions of traditional psychosocial interventions are usually limited to simple support groups and patient–provider communication [57]. Valente (2012) describes in detail four strategies for social network interventions: (1) identifying individuals to act as agents/catalysts for improving interactions of the network members, (2) involving groups of people in the network intervention, (3) fostering new interactions among people, and (4) changing the existing network by incorporating the dynamic nature of social network activities [58]. Social network interventions provide practical methods for enhancing social networks, which may be more sustainable than traditional psychosocial interventions, when cancer patients and survivors finish the intervention and carry on in their normal lives [59]. Social network interventions have been implemented most often to target sexual health and HIV prevention, yet they have been used seldom with cancer populations [60]. A recent intervention in cancer survivors with significant distress used multiple mHealth platforms (e.g., discussion board, chat, email, blog) and reported increased social engagement among participants in the discussion board group versus other groups, although this study was excluded from the current review due to the survivors’ older age (50 years or above) [61]. For a social network intervention to be implemented appropriately in cancer patients and survivors, unique characteristics of the participants need to be considered, such as the time since cancer diagnosis; the type of cancer; and/or treatment-associated late effects, including cognitive limitations [62] and hearing or vision loss [63].

## 5. Limitations

This systematic review study has several limitations. First, we only included studies that focused on young- and middle-aged cancer patients and/or survivors, which limits the generalizability to cancer populations across all ages. We excluded individuals in late adulthood aged over 65 years because their social interactions (e.g., having adult children and/or grandchildren), health experiences (e.g., increased chronic health conditions typical of aging), and life satisfaction are usually different than young- and middle-aged adults [15,64,65]. Second, most cited studies focused on breast cancer patients, although our initial purpose was to identify articles containing patients/survivors diagnosed with any cancer type in young- or middle-aged adulthood for evaluating the effects of social integration or connectedness interventions. Therefore, the generalizability of our findings to all types of cancers is limited, and differences across cancer subgroups (e.g., breast vs. other cancers) may not be comparable. Future interventional studies focusing on subgroups of cancer patients/survivors beyond breast cancer are warranted to evaluate the effects of social integration or connectedness on social and health-related outcomes. Third, we could not perform a meta-analysis to draw concrete conclusions due to a lack of homogenous designs and endpoints in the studies included in our review. Only a few studies reported effect sizes for the effects of the intervention on social and health-related outcomes, and the magnitudes of the effect size varied, ranging from small to large effects. The primary aim of most included studies was to address psychosocial impairment, rather than social connectedness or integration problems in cancer populations, and they did not include impaired social integration as a criterion to enroll participants, although two studies did assess perceived isolation at baseline [28,44].

## 6. Conclusions

Social integration interventions that adopt technology- and/or non-technology-based platforms show improved social and emotional support, decreased perceived isolation, increased knowledge and access to cancer survivorship resources, and better PROs in young- and middle-aged adult cancer patients and survivors versus the control counterparts. Based on personal scheduling/availability, geographical restriction, and disease characteristics, using appropriate technology- and/or non-technology-based platforms to connect cancer patients/survivors to friends, cancer peers, or members of society to obtain needed resources and support can help patients and survivors cope with stressful life circumstances and optimize social and health outcomes. Novel social network interventions based on a strong theoretical framework are needed to address the dynamic and complex nature of social integration in cancer patients and survivors.

## Figures and Tables

**Table 1:** Characteristics of the included studies.

Author (Publication Year)	Country	Study Aim	Study Sample [Survivors/Patients, Diagnoses, Sample Sizes]	Age in Years (Mean ± SD)	Sex	Race/Ethnicity	Time Since Cancer Diagnosis in Years, Months, or Days (Mean ± SD)	Intervention Platform	Outcome of Interest
Technology-based interventions									
Ashing-Giwa (2008) [26]	USA	Assess the feasibility of implementing culturally sensitive telephone intervention for Latina American cervical cancer survivors.	Survivors Diagnosis: Cervical Sample sizes: IG = 15 CG = 8	IG = 47.9 ± 6.9 CG = 55.5 ± 14.9	Female = 23	Latina American = 23	IG = 3.0 ± 1.2 years CG = 4.1 ± 1.5 years	Telephone	PRO (QOL)
Casillas (2019) [27]	USA	Compare text messaging and peer navigation to traditional and standard-of-care online materials (Health Links) to inform adolescents and young adults on cancer survivorship care.	Survivors Diagnoses: Leukemia, lymphoma, CNS, kidney, bone/soft-tissue sarcoma Sample sizes: IG (text messaging) = 28 IG (peer navigation) = 25 CG = 25	IG (text messaging) = 21 ± 5 IG (peer navigation) = 21 ± 6 CG = 20 ± 5	IG (text messaging): Male = 15 Female = 13 IG (peer navigation): Male = 11 Female = 14 CG: Male = 11 Female = 14	IG (text messaging): Non-Hispanic/Latino White = 11; Black = 1; Asian = 3; Hispanic/Latino = 12; Mixed race/ethnicity = 1 IG (peer navigation): Non-Hispanic/Latino White = 10; Hispanic/Latino = 11; Mixed race/ethnicity = 4 CG: Non-Hispanic/Latino White = 8; Black = 1; Asian = 1; Hispanic/Latino = 13; Mixed race/ethnicity = 2	Time since completing treatment: IG (text messaging) = 8 ± 5 years IG (peer navigation) = 9 ± 7 years CG = 8 ± 7	Telephone	PROs (knowledge, attitudes, self-efficacy for survivorship care planning)
Chee (2020) [28]	USA	Explore preliminary efficacy of technology-based cancer pain management support program for Asian American survivors of breast cancer in improving cancer pain experience.	Survivors Diagnosis: Breast Sample sizes: IG = 64 CG = 30	IG = 38.4 ± 6.20 CG = 48.0 ± 11.1	Female = 94	IG: Chinese = 63; Other = 1 CG: Chinese = 13; Korean = 6; Japanese = 3; Other = 5	IG = 2.5 ± 1.2 years CG = 1.1 ± 0.6 years	Online discussion board, online educational sessions, online resources including videos	Social outcome (isolation), PROs (pain, uncertainty, self-efficacy), and health-related resources (cancer-related health resources)
Classen (2012) [41]	Canada	Examine participation rates and preliminary outcomes for online support group after gynecologic cancer treatment.	Patients Diagnoses: Cervical, ovarian, uterine Sample sizes: IG = 13 CG = 14	IG = 39.9 CG = 44.6	Female = 27	IG: Black = 2; Asian = 1; White/European = 10 CG: Asian = 2; Latin American = 2; White/European = 10	Time since completing treatment: IG = 24.3 ± 10.4 months CG = 31.3 ± 26.7 months	Online synchronous and asynchronous sessions	PROs (sexual distress, depression, anxiety, illness intrusiveness)
Gustafson (2001) [29]	USA	Assess the impact of a computer-based patient support system on QOL in younger women with breast cancer.	Patients Diagnosis: Breast Sample sizes: IG = 121 CG = 125	IG = 44.3 ± 6.6 CG = 44.4 ± 7.1	Female = 246	IG: Caucasian = 92; Other = 29 CG: Caucasian = 90; Other = 35	IG = 55.0 ± 36.3 days CG = 47.4 ± 32.6 days	Home computer through the central server for communication	Social outcomes (communication with healthcare providers, social support) and PRO (QOL)
Lang (2020) [42]	Canada	Examine online synchronous chat group-plus-education (OSG + E) and online synchronous chat group-using video (OSG + V) models’ content sustainability, group processes, and feasibility in adolescent and young adult cancer survivors.	Survivors Diagnoses: Hematopoietic, breast, CNS, digestive organs Sample sizes: IG (OSG + E) = 8 IG (OSG + V) = 8 CG = 18	IG (OSG + E) = 34.5 ± 4.6 IG (OSG + V) = 28.9 ± 4.3 CG = 29.8 ± 5.8	IG (OSG + E): Male = 2 Female = 6 IG (OSG + V): Male = 3 Female = 5 CG: Male = 6 Female = 12	Not reported	IG (OSG + E) = Median 21.5 months IG (OSG + V) = Median 7.5 months CG = Median 8.0 months	Online chat and video	Social outcomes (group cohesion, valued group experiences) and PROs (post-traumatic stress and growth, loneliness, distress, coping)
Valle (2013) [31]	USA	Evaluate adherence to and acceptability of Facebook-based intervention approaches to improve moderate-to-vigorous physical activity.	Survivors Diagnoses: Hematologic, breast, gynecologic, head and neck, gastrointestinal, musculoskeletal, genitourinary, lung Sample sizes: IG = 45 CG = 41	IG = 30.8 ± 5.7 CG = 32.7 ± 4.2	IG: Male = 4 Female = 41 CG: Male = 4 Female = 37	IG: Non-Hispanic White = 42; Black/other = 3 CG: Non-Hispanic White = 36; Black/other = 5	IG = 63.2 ± 7.8 months CG = 53.7 ± 5.1 months	Facebook group, videos	PRO (QOL) and lifestyle outcome (physical activity)
Valle (2015) [32]	USA	Evaluate (1) the effects of physical activity intervention for young adult cancer survivors on changes in self-efficacy, social support, and self-monitoring for behavior change; (2) whether changes in social cognitive theory constructs mediated the relationship between intervention and changes in physical activity.	Survivors Diagnoses: Excluded non-melanoma skin cancer Sample sizes: IG = 45 CG = 41	IG = 30.8 ± 5.7 CG = 32.7 ± 4.2	IG: Male = 4 Female = 41 CG: Male = 4 Female = 37	IG: Non-Hispanic White = 42; Black/other = 3 CG: Non-Hispanic White = 36; Black/other = 5	Not reported	Facebook group, videos	Social outcome (social support), PRO (self-monitoring), and lifestyle outcome (physical activity)
Valle (2017) [30]	USA	Evaluate the use of Facebook for engaging in a social networking site-based physical activity intervention program among young adult cancer survivors.	Survivors Diagnoses: Excluded non-melanoma skin cancer Sample sizes: IG = 45 CG = 41	IG = 30.8 ± 5.7 CG = 32.7 ± 4.2	IG: Male = 4 Female = 41 CG: Male = 9 Female = 37	IG: Non-Hispanic White = 42; Black/other = 3 CG: Non-Hispanic White = 36; Black/other = 5	IG = 63.2 ± 52.1 months CG = 52.7 ± 32.7 months	Facebook group, videos	Lifestyle outcome (physical activity)
Winzelberg (2003) [33]	USA	Evaluate the psychological benefits of online breast cancer support groups.	Patients Diagnosis: Breast Sample sizes: IG = 36 CG = 36	Total = 49.5 ± 6.2	Female = 72	Caucasian = 58; African American = 3; Asian = 3; Hispanic/Latina = 4; Other = 4	12 ± 9 months	Online support group	PROs (depression, stress, trauma)
Zhou (2020) [38]	China	Evaluate benefits of mobile internet-based communication software (WeChat) multimodal nursing program on early rehabilitation in post-operative women with breast cancer.	Patients Diagnosis: Breast Sample sizes: IG = 56 CG = 55	IG = 49.84 ± 8.85 CG = 49.98 ± 9.84	Female = 111	Not reported	Not reported	WeChat (mobile internet-based communication software)	PRO (QOL)
Non-technology-based interventions									
Chan (2006) [52]	Hong Kong	Evaluate effects of psychosocial interventions on psychophysiological outcomes in breast cancer patients.	Patients Diagnosis: Breast Sample sizes: IG (Body–Mind–Spirit) = 27 IG (supportive expressive) = 16 IG (social support self-help) = 16 CG = 17	IG (Body–Mind–Spirit) = 49.5 ± 6.9 IG (supportive expressive) = 46.9 ± 8.8 IG (social support self-help) = 50.3 ± 8.4 CG = 47.5 ± 9.8	Female = 76	Chinese = 76	IG (Body–Mind– Spirit) = 22.9 ± 17.3 months IG (supportive expressive) = 29.1 ± 24.0 months IG (social support self-help) = 28.8 ± 14.8 months CG = 29.9 ± 16.9 months	In-person sessions	Social outcome (social support) and PROs (emotional control, stress)
Classen (2008) [35]	USA	Evaluate the effectiveness of a supportive–expressive group therapy program among breast cancer patients treated in community settings and determine whether highly distressed patients were most likely to benefit.	Patients Diagnosis: Breast Sample sizes: IG = 177 CG = 176	IG = 49.8 ± 10.9 CG = 49.7 ± 10.6	Female = 353	IG: Black = 4; Asian American = 3; Native American = 5; White/European American = 162 CG: Black = 6; Asian American = 4; Mexican American = 2; Other Hispanic/Latina = 1; Native American = 11; White/European American = 149	IG = 7.5 ± 3.8 months CG = 6.9 ± 3.7 months	In-person sessions	Social outcomes (family relations, social support) and PROs (mood, anxiety, depression)
Gonzales (2016) [36]	USA	Evaluate the relationship between emotional social support and emotional well-being in Latina immigrant women with breast cancer.	Patients Diagnosis: Breast Sample size: Total = 150	Total = 50.1 ± 10.9	Female = 150	Region of origin: Mexico = 101; Central America = 35; South America = 14	Diagnosed in the past year	In-person sessions	Social outcome (emotional social support) and PROs (emotional well-being, acceptance)
Hoffman (2012) [49]	UK	Evaluate the effectiveness of mindfulness-based stress reduction on mood, breast- and endocrine-specific QOL, and well-being post-treatment in women with stages 0 to III breast cancer.	Patients Diagnosis: Breast Sample sizes: IG = 114 CG = 115	IG = 49 ± 9.26 CG = 50.1 ± 9.14	Female = 229	Not reported	IG = 17.4 ± 13 months CG = 19.0 ± 15 months	In-person sessions	Social outcome (social well-being) and PROs (anxiety, mood, depression, anger, fatigue, well-being)
Kissane (2004) [50]	Australia	Evaluate the impact of cognitive–existential group therapy on survival in women with early-stage cancer.	Patients Diagnosis: Breast Sample sizes: IG = 154 CG = 149	IG = 45.4 ± 8.0 CG = 47.3 ± 8.3	Female = 303	Not reported	Total = 102 ± 56 days	In-person sessions	PROs (anxiety, family functioning) and survival outcome
Samami (2020) [43]	Iran	Investigate the effect of the supportive program on coping strategies and stress in women with breast cancer.	Patients Diagnosis: Breast Sample sizes: IG = 27 CG = 30	IG = 43.8 ± 7.4 CG = 44.0 ± 7.3	Female = 57	Not reported	IG = 4.6 ± 2.0 months CG = 5.4 ± 1.5 months	In-person sessions	PROs (coping, stress)
Scheier (2005) [34]	USA	Evaluate effects of educational intervention and nutritional intervention on enhancing physical and psychological functioning in younger women with early-stage breast cancer.	Patients Diagnosis: Breast Sample sizes: IG (nutrition) = 78 IG (education) = 70 CG = 76	IG (nutrition) = 44.2 IG (education) = 43.7 CG = 44.6	Female = 224	IG (nutrition): Caucasian = 71; African American = 4; Other = 3 IG (education): Caucasian = 67; African American = 3; Other = 0 CG: Caucasian = 74; African American = 2; Other = 0	IG(nutrition) = 6 months IG(education) = 6 months CG = 6.5 months	In-person sessions	PROs (depression, QOL)
Schover (2006) [37]	USA	Describe the use of a peer counseling program to improve sexual function and reproductive health and decrease menopausal symptoms and infertility-related distress for African American breast cancer survivors.	Survivors Diagnosis: Breast Sample size: Total = 48	Total = 49.3 ± 8.4	Female = 48	African American = 48	Total = 4.5 ± 3.8 years	In-person sessions	PROs (reproductive issues, emotional distress)
Sturm (2014) [51]	Germany	Evaluate the effect of dance as a holistic sportive activity in cancer patients under active cancer treatment.	Patients Diagnoses: Breast, ovarian, gastrointestinal Sample sizes: IG = 20 CG = 20	IG = 49.0 (median) CG = 50.5 (median)	IG: Male = 1 Female = 19 CG: Male = 2 Female = 18	Not reported	Not reported	In-person sessions	Social outcome (social functioning) and PROs (fatigue, emotional functioning, QOL)
Tabrizi (2016) [44]	Iran	Evaluate the effect of supportive–expressive discussion groups on loneliness, hope, and QOL in breast cancer survivors.	Patients Survivors: Breast Sample sizes: IG = 41 CG = 40	Total = 47.9 ± 11.4	Female = 81	Not reported	Total (frequency): <6 months since diagnosis = 11 >6 months since diagnosis = 70	In-person sessions	Social outcome (loneliness) and PROs (QOL, hope, mental health)
Vos (2004) [45]	Netherlands	Examine the effects of two psychosocial intervention programs in women with primary, non-metastatic breast cancer.	Patients Diagnosis: Breast Sample sizes: IG (group psychotherapy) = 15 IG (social support) = 19 CG = 35	Total = 49.2 ± 7.90	Female = 69	Not reported	Total = 9.3 weeks	In-person sessions	Social outcome (social support) and PROs (coping, mood)
Vos (2007) [46]	Netherlands	Evaluate the effectiveness of experiential–existential group psychotherapy or social support group on psychosocial adjustment in women with primary breast cancer.	Patients Diagnosis: Breast Sample sizes: IG (group psychotherapy) = 33 IG (social support) = 34	IG (group psychotherapy) = 49.0 IG (social support) = 49.4	Female = 67	Not reported	Time since surgery: IG (group psychotherapy) = 10.7 weeks IG (social support) = 13.0 weeks	In-person sessions	Social outcome (social interactions), PROs (mood, body image), and lifestyle outcome (recreation)
Zhang (2017) [39]	China	Evaluate the efficacy of mindfulness-based stress reduction on promoting post-traumatic growth and decreasing perceived stress and anxiety in Chinese breast cancer patients.	Patients Diagnosis: Breast Sample sizes: IG = 30 CG = 30	IG = 48.7 ± 8.5 CG = 46.0 ± 5.1	Female = 60	Not reported	Not reported	In-person sessions	PROs (stress, anxiety)
Mixed-type (technology- and non-technology-based) interventions									
Chen (2019) [53]	Taiwan	Evaluate the effect of the behavior change program and health education on depression, fear of social interactions, avoidance of social interactions, physical function, and social–emotional function in head and neck cancer survivors.	Survivors Diagnoses: Head and neck Sample sizes: IG = 50 CG = 50	IG = 47.8 ± 1.0 CG = 49.1 ± 1.1	IG: Male = 40 Female = 10 CG: Male = 41 Female = 1	Not reported	IG = 3.0 ± 1.3 months CG = 3.1 ± 1.2 months	In-person sessions, provided video disc to watch after session, telephone	Social outcome (social interactions) and PROs (depression, anxiety, QOL)
Lee (2013) [47]	South Korea	Test effects of a dyadic peer support intervention on self-efficacy, anxiety, depression, and mental adjustment among newly diagnosed breast cancer patients in Korea.	Patients Diagnosis: Breast Sample sizes: IG = 64 CG = 65	Total = 47.7 ± 7.0	Female = 129	Not reported	Total = within 1 month of diagnosis	In-person sessions and telephone	PROs (anxiety, depression, self-efficacy, mental adjustment)
Park (2012) [48]	South Korea	Examine the effect of a psychoeducational support program on QOL and symptoms in women with breast cancer one-year post-treatment.	Survivors Diagnosis: Breast Sample sizes: IG = 25 CG = 23	IG = 44.3 ± 6.0 CG = 47.6 ± 6.2	Female = 48	Not reported	Not reported	In-person sessions and telephone	PROs (QOL, emotional well-being)
Ye (2016) [40]	China	Examine the efficacy of a multidiscipline mentor-based program on 1) increased protective factors of social support and hope for the future; 2) decreased risk factors of physical and emotional distress; 3) improved resilience, transcendence, and QOL.	Patients Diagnosis: Breast Sample sizes: IG = 93 CG = 82 Norm group = 76	Frequencies: IG (≤30 years) = 17 IG (31 to ≤50 years) = 35 IG (>50 years) = 41 CG (≤30 years) = 12 CG (31 to ≤50 years) = 37 CG (>50 years) = 33 Norm group (≤30 years) = 21 Norm group (31 to ≤50 years) = 32 Norm group (>50 years) = 23	Female = 251	IG: Han = 89; Other = 4 CG: Han = 77; Other = 5 Norm group: Han = 72; Other = 4	Not reported	In-person sessions and telephone	Social outcome (social support) and PROs (QOL, depression, hope)

Note: IG = intervention group; CG = control group; PROs = patient-reported outcomes; QOL = quality-of-life.

**Table 2:** Technology-based interventions, social integration component of the intervention, results, and implications of the interventions.

Author (Year)	Intervention(s) Description	Control Group (CG) Description	Intervention Duration	Social Cognitive Theory Component of the Intervention	Results	Implication
Ashing-Giwa (2008) [26]	During telephone counseling sessions, clinical research associates guided survivors in discussing strategies to address individual survivors’ specific concerns across eight domains: orientation and assessment, health education and referrals, coping skills/problem-solving, balancing emotions and stress management and relaxation, family communication skills, relational/sexual communication skills, social support network, and contextual reinforcement and debriefing.	Provided reading materials that included information about cervical cancer, sexuality, stress management, communication with doctors, family communication, information on clinical trials and nutrition, and psychological and medical resources.	12 weeks	Coping (affect), practice new skills in or outside the intervention, helping set realistic expectations, problem solving	Increased physical well-being (*p* = 0.045) and overall QOL (*p* = 0.045) versus controls. Increased awareness of psychological and medical resources, communication, and utilization of healthcare resources.	Improving physical and overall QOL in low-income Latina American cervical cancer survivors is feasible through individually tailored counseling.
Casillas (2019) [27]	The text messaging intervention group received an educational book, chose the goals per Adolescent and Young Adult Survivorship Action Plan (ASAP) after reading the book, and texted the goals to the provided number for initiating two-way automated communication to support survivor engagement in accessing community and cancer center resources. The peer-navigation intervention group received an educational book, chose ASAP goals after reading the book, then was matched with a peer navigator. Peer navigators contacted participants through two telephone calls and reviewed ASAP goals and discussed community and cancer center resources to achieve goals.	Provided Health Links (standard of care educational materials created by the Children’s Oncology Group) in the mail after study enrollment. After receiving Health Links, another piece of paper was mailed asking participants to formulate their ASAP goals and develop strategies to help them achieve their ASAP goals. They were encouraged to use the Health Links educational material to find answers and achieve their goals by discussing with their healthcare provider during the study period.	8 weeks	Goal setting, self-help materials (bibliotherapy)	Text messaging group: higher survivorship care knowledge versus controls (*p* < 0.05, Cohen’s d = 0.70). Peer-navigation group: higher survivorship care planning self-efficacy (*p* < 0.05, Cohen’s d = 0.68), higher late effects self-efficacy (*p* < 0.05, Cohen’s d = 0.65), and higher health insurance self-efficacy (*p* < 0.05, Cohen’s d = 0.47) versus controls. Both the text messaging group (*p* < 0.05, Cohen’s d = 0.33) and peer navigation group (*p* < 0.05, Cohen’s d = 0.37) had a better attitude in seeking survivorship care versus controls.	Offering cost-effective and convenient communication via the telephone can help to improve survivorship care.
Chee (2020) [28]	Participants were provided access to the cancer pain management support program for Asian American survivors of breast cancer, a technology-based cancer pain management program that provided interactive online discussion and resources, including videos from scientific authorities, such as the National Institute of Health and American Cancer Society. Participants posted breast cancer experiences and received individual and group support/coaching from culturally matched participants and healthcare providers.	Provided the website of American Cancer Society. After the intervention and post-assessment were completed, control group participants were offered the support and coaching that was provided to the intervention group.	4 weeks	Coping (affect), cancer survivor/patient testimony, self-help materials (bibliotherapy)	No significant difference in cancer-related pain between the groups. Decrease in perceived isolation (*p* < 0.01) and the degree of uncertainty (*p* < 0.01) and increase in personal resources (*p* < 0.05) in intervention groups versus controls.	Technology-based culturally tailored intervention is feasible for Asian American breast cancer survivors.
Classen (2012) [41]	Moderators introduced a topic each week and asked questions to facilitate the discussion. In week 10, a 90 min text-based chat session (i.e., synchronous session) was offered, in which participants interacted with a gynecologic oncologist, radiation oncologist, and moderators. Asynchronous components were discussion forums facilitated by cancer support groups, online support groups, and supportive group psychotherapy for psychosexual concerns. Participants interacted by posting messages, responding to questions, and involvement in the discussion. Cancer-related educational materials and links to online resources were also provided.	Control groups were assigned as a wait-list control group. Wait-list controls were offered intervention after the intervention group completed the intervention.	12 weeks	Coping (affect), cancer survivor/patient testimony, self-help materials (bibliotherapy)	Of 21 respondents, 12 (57%) felt more comfortable discussing sexual issues via a web-based support group versus a face-to-face group, and 6 (29%) were less comfortable. Intent-to-treat analyses suggest a small effect for a reduction in sexual distress. Intervention enhanced intimacy, but this was not statistically significant.	Web-based support group intervention is feasible for addressing psychosexual concerns.
Gustafson (2001) [29]	To address the needs identified by cancer patients, the intervention included 11 services in three categories: 1) information services—questions/answers, instant library, consumer guide, and referral directory; 2) support services—discussion groups (sharing information and support), ask experts, share personal stories; 3) decision services—assessment (emotional status and coping advice), health charts, decision aid, action plans (identifying goals, resources, and ways to overcome obstacles). Participants accessed materials at their discretion.	Participants in the control group received a copy of Dr. Susan Love’s Breast Book as information regarding breast cancer.	26 weeks	Coping (affect), goal setting, cancer survivor/patient testimony, self-help materials (bibliotherapy)	After 2 months, the intervention group had higher information competence (*p* < 0.01), a higher level of comfort participating in the intervention (*p* < 0.01), and higher confidence communicating with doctors regarding their healthcare (*p* < 0.05) versus the control. After 5 months, the intervention group had higher social support [*p* < 0.01] and greater information competence [*p* = 0.05] versus the control.	Computer-based support, among those who have Internet access, can benefit breast cancer patients by providing information and social support.
Lang (2020) [42]	The online synchronous chat group-plus-education intervention model (OSG + E) focused on teaching coping skills (i.e., mindfulness, relaxation, communication, social support, sexuality, and healthy lifestyle) through real-time text conversations. The online synchronous chat intervention group, using a video, model (OSG + V) shared a video to initiate group discussions and focused on sharing feelings, building connections, and cultivating supportive peer relationships.	Participants assigned to the wait-list control group.	10 weeks	Relaxation training, coping (affect), cancer survivor/patient testimony, problem solving	Both OSG + E and OSG + V showed improvement in group cohesion versus controls; however, the participants rated OSG + V as more suitable, cohesive, and having higher levels of important group processes.	The use of digital storytelling tools to stimulate discussion, foster a sense of belonging, and convey information is feasible.
Valle (2013) [31]	Participants wore a pedometer, received physical activity goals, and were added to a Facebook group, through which they received daily messages with physical activity information. The intervention group received additional messages to initiate group interaction and social support within the Facebook group. The study moderator posted prompts to the Facebook group, including (1) discussion questions; (2) links to videos, cancer-related news articles, and electronic physical activity resources; and (3) weekly reminders to set up exercise goals, log daily physical activity, and check out posted resources on the Facebook group.	Participants received pedometer in the mail with instructions on how to use it and record their total daily steps. Participants received an introductory email regarding the goal and recommendation regarding physical activity. Participants received links to publicly available websites related to physical activity and/or cancer survivorship, 12 weekly Facebook messages with basic information on physical activity, assigned Facebook group, no access to self-monitoring website, and were not prompted to interact within their Facebook group.	12 weeks	Coping (physical), self-monitoring of skills/thoughts/etc., goal setting, cancer survivor/patient testimony, self-help materials (bibliotherapy)	Both intervention (*p* = 0.009) and control group (*p* = 0.045) showed increase in self-reported weekly moderate-to-vigorous physical activity with no significant difference between groups (*p* = 0.549). Increase in light physical activity (*p* = 0.032) was observed in the intervention group versus controls.	Social networking sites are a feasible way to distribute health information and support to promote physical activity and healthy behaviors in cancer survivors.
Valle (2015) [32]	Participants wore a pedometer, received physical activity goals, and were added to a Facebook group, through which they received daily messages with physical activity information. The intervention group received additional messages to initiate group interaction and social support within the Facebook group. The study moderator posted prompts to the Facebook group, including (1) discussion questions; (2) links to videos, cancer-related news articles, and electronic physical activity resources; and (3) weekly reminders to set up exercise goals, log daily physical activity, and check out posted resources on the Facebook group.	Participants received pedometer in the mail with instructions on how to use it and record their total daily steps. Participants received an introductory email regarding the goal and recommendation regarding physical activity. Participants received links to publicly available websites related to physical activity and/or cancer survivorship, 12 weekly Facebook messages with basic information on physical activity, assigned Facebook group, no access to self-monitoring website, and were not prompted to interact within their Facebook group.	12 weeks	Coping (physical), self-monitoring of skills/thoughts/etc., goal setting, cancer survivor/patient testimony, self-help materials (bibliotherapy)	Intervention group had lower self-efficacy for adhering to physical activity (*p* = 0.025) and social support from friends on social networking sites (*p* = 0.039) versus controls. Intervention group showed a positive effect on mild physical activity. Changes in social support on social networking sites partially mediated the intervention effects on moderate-to-vigorous physical activity in an unexpected direction.	Researchers can target social cognitive theory constructs, including social support, to promote physical activity in cancer survivors.
Valle (2017) [30]	Participants wore a pedometer, received physical activity goals, and were added to a Facebook group, through which they received daily messages with physical activity information. The intervention group received additional messages to initiate group interaction and social support within the Facebook group. The study moderator posted prompts to the Facebook group, including (1) discussion questions; (2) links to videos, cancer-related news articles, and electronic physical activity resources; and (3) weekly reminders to set up exercise goals, log daily physical activity, and check out posted resources on the Facebook group.	Participants received pedometer in the mail with instructions on how to use it and record their total daily steps. Participants received an introductory email regarding the goal and recommendation regarding physical activity. Participants received links to publicly available websites related to physical activity and/or cancer survivorship, 12 weekly Facebook messages with basic information on physical activity, assigned Facebook group, no access to self-monitoring website, and were not prompted to interact within their Facebook group.	12 weeks	Coping (physical), self-monitoring of skills/thoughts/etc., goal setting, cancer survivor/patient testimony, self-help materials (bibliotherapy)	Intervention group reported that group discussions caused them to become less physically active (*p* = 0.040) and felt that group members were less supportive (*p* = 0.028) versus controls. Responses posted on Facebook increased light physical activity (*p* = 0.049) across groups.	Peer-led discussions through Facebook help improve group interactions and may be associated with physical activity among cancer survivors.
Winzelberg (2003) [33]	The intervention was moderated by a mental health professional to facilitate discussion. Asynchronously, participants described illness and treatment and interacted with others by sharing their thoughts openly and honestly, emotional situations, success and frustration, feelings of uncertainty and strategies for coping with feelings about self/body images, romance and sexuality, relationship with family/friends, fear of recurrence, and meaning of life including changes in priorities.	Participants in the wait-list control group were invited to participate in their own support after the post-treatment assessment.	12 weeks	Coping (affect), coping (spiritual), self-monitoring of skills/thoughts/etc., cancer survivor/patient testimony	Intervention group showed reductions in depression (effect size = 0.54, *p* < 0.01), cancer-related trauma (effect size = 0.45, *p* < 0.01), and perceived stress (effect size = 0.37, *p* < 0.05) versus controls.	Cancer patients benefit from receiving open and honest experiences from peers through web-based communications.
Zhou (2020) [38]	In stage I (from hospital admission to surgery) (a) physical rehabilitation focused on the provision of individualized information (e.g., illness condition, diet, rest) and appropriate exercise training; (b) psychological rehabilitation on feeling expression, communicating with relatives and significant others or peers; (c) social rehabilitation on adaptation to the patient role and social training (e.g., avoiding social isolation). In stage II (post-surgery) (a) physical rehabilitation on recurrence prevention, and coping; (b) psychological counseling, peers sharing coping strategies; and (c) social rehabilitation on role transformation from patient to social role.	Participants in the control group received routine nursing care, such as health education, monitoring vital signs, and post-operative complications, and post-operative and drainage tube care.	6 months	Relaxation training, coping (physical), coping (affect), self-monitoring of skills/thoughts/etc., goal setting	Physical well-being exhibited a time effect (*p* < 0.001). Social/family well-being had group (*p* < 0.001), time (*p* < 0.001), and group–time interaction (*p* = 0.01) effects. Emotional well-being had time (*p* < 0.001) and group–time interaction effects (*p* < 0.001). Functional well-being had group (*p* < 0.001), time (*p* < 0.001), and group–time interaction (*p* = 0.004) effects.	WeChat-based multimodal nursing program can improve the well-being of breast cancer patients who have recently undergone surgery.

Note: QOL = quality-of-life. In all interventions, participants were randomly assigned to intervention or control groups.

**Table 3:** Non-technology-based interventions, social integration component of the intervention, results, and implications of the interventions.

Author (Year)	Intervention(s) Description	Control Group (CG) Description	Intervention Duration	Social Cognitive Theory Component of the Intervention	Results	Implication
Chan (2006) [52]	Body–Mind–Spirit (BMS) intervention group integrated western psychotherapeutic elements into eastern/Chinese health practices (e.g., normalizing traumatic experiences, forgiveness) and used group therapy to explore different ways of learning and expression (e.g., reading, writing, singing, physical activity, meditation, and drawing). Supportive–Expressive (SE) intervention group (western style approach) was led by clinical psychologists and a medical social worker through group therapy sessions focused on building new bonds of social support, dealing with fears of death and body images, reordering life priorities, improving relationships with family/friends, and communicating with healthcare providers. The social support self-help (SS) intervention group (non-professional-led group) did not have a structured program, but a social worker encouraged the group to gather and communicate with people outside the group.	Participants in the control group received educational materials, such as, information regarding nutrition, diet, dealing with edema on arms, body care after chemo- and radiation-therapy.	8 weeks	Coping (affect), coping (spiritual), cancer survivor/patient testimony	BMS intervention group after 4 months had a reduction in general health (*p* < 0.05) and higher positive support (*p* < 0.05), and after 8 months, had reductions in emotional control (*p* < 0.05) and negative emotions (*p* < 0.05) versus controls. SS intervention group after 4 months had higher negative emotions (*p* < 0.05) versus controls. SE intervention group did not show statistically significant changes at 4 or 8 months compared to baseline.	Intervention in a culturally supportive and encouraging environment with active professional involvement yields therapeutic effects.
Classen (2008) [35]	Educational materials and pamphlets published by the American Cancer Society were mailed to participants. The intervention group received supportive–expressive group therapy with therapists, which was an unstructured intervention designed to build new bonds of social support, expression of emotion, enhance communication, enhance symptom control, and deal with concerns, such as fears of death, changes in self/body image, making meaning out of illness, feelings of isolation, and reordering life priorities.	Participants in the control group received educational materials and a brief videotape on breast self-examination as well as pamphlets published by the American Cancer Society in their mail. The pamphlets included facts on cancer, breast self-examination, cooking, helping children understand sexuality, radiation, chemotherapy, and breast changes, but no information on emotional expression or social support.	12 weeks	Coping (affect), coping (spiritual), self-help materials (bibliotherapy)	No significant benefit from the supportive–expressive group therapy. When an outlier with very high distress was included in the analysis, the intervention group had lower anxiety (*p* = 0.034), depression (*p* = 0.021), helplessness/hopelessness (*p* = 0.024), negative support (*p* = 0.044), and higher instrumental support (0.020) versus controls.	Supportive–expressive group therapy did not benefit highly distressed cancer individuals.
Gonzales (2016) [36]	A trained counselor (who was also a breast cancer survivor) met with participants eight times in person over the 8 weeks. The intervention provided cognitive–behavioral coping skills in managing the initial impact of cancer, finding cancer information (effective communication with healthcare providers), getting needed support (talking about cancer with others, identifying sources of support, asking for needed help), thoughts and mood (identifying and managing stress and its causes and coping), stress management techniques, and setting goals for future. Participants were also provided with a list of cancer-related local community resources for support.	Participants in the control group were offered the intervention after 6 months, i.e., after completion of assessment.	8 weeks	Coping (affect), cognitive restructuring, goal setting, self-help materials (bibliotherapy)	Emotional support and acceptance of cancer were positively associated with emotional well-being (*p* = 0.001), whereas fatalism was negatively associated with emotional well-being (*p* < 0.001). Significant direct effect of emotional support on emotional well-being (b = 0.88, 95% CI: 0.24, 1.52). Significant indirect effect of emotional support on emotional well-being through fatalism (b = 0.21, 95% CI: 0.04, 0.51) and a marginally significant indirect effect through acceptance (b = 0.15, 95% CI: 0.001, 0.43).	Emotional support may increase well-being in Latina cancer survivors.
Hoffman (2012) [49]	Mindfulness-based stress reduction classes focused on the practice of a body scan, gentle and appropriate lying and standing yoga-based stretches, sitting meditation, some group discussions, didactic teaching, and home practice on topics including perceptions of and reactions to life events, stress physiology, and mindfulness in communication and everyday life. Participants were also asked to practice at home for 40-45 min for 6 or 7 days per week.	Participants in the wait-list control group continued with their lives as usual. They were offered measurement tools.	8 weeks	Relaxation training, practice new skills in or outside the intervention, self-monitoring of skills/thoughts/etc.	Participants in the intervention group had significant reductions in mood (*p* < 0.001), anxiety (*p* < 0.001), depression (*p* = 0.017), anger (*p* = 0.005), vigor (*p* < 0.001), fatigue (*p* = 0.002), and confusion (*p* = 0.002) versus controls. Significant treatment effects of intervention for physical (*p* = 0.002), social (*p* = 0.032), emotional (*p* = 0.001), functional (*p* < 0.001), and overall (*p* < 0.001) well-being versus controls.	Mindfulness-based stress reduction intervention can improve mood, QOL, and well-being in breast cancer patients.
Kissane (2004) [50]	Six to eight participants met with two therapists in group therapy sessions. Therapy-related goals were promoting a supportive environment, reframing negative thinking, enhancing coping problem-solving, fostering hope, and setting priorities for the future. The group shared their experience of illness. Women exchanged phone numbers and met outside informally for support.	Participants in the control group received three relaxation classes, but no weekly group therapy.	20 weeks	Relaxation training, coping (affect), coping (spiritual), practice new skills in or outside the intervention, cognitive restructuring, goal setting, problem solving	Reduced anxiety (*p* = 0.05), improved family functioning (*p* = 0.07), and greater satisfaction with therapy (*p* < 0.001) in intervention groups versus controls. Median survival was 81.9 months (95% CI: 64.8, 99.0) in the intervention group compared to 85.5 months (95% CI: 67.5, 103.6) in controls. The hazard ratio for death in the intervention group was 1.35 (*p* = 0.31, 95% CI: 0.76, 2.39) versus controls. Intervention group achieved significant cohesiveness, and they continued to meet regularly for several years after the intervention.	Cognitive–existential group therapy had effects on psychosocial outcomes, but did not prolong survival, in women with early-stage breast cancer.
Samami (2020) [43]	Ten participants were in each group session and were provided education about breast cancer, cancer-related stress and management, and problem and emotional coping strategies. Participants discussed information about social support and coping strategies.	Participants received routine care, which included trainings related to the post-chemotherapy physical and nutritional problems.	6 weeks	Relaxation training, coping (physical), coping (affect), coping (spiritual), practice new skills in or outside the intervention	Problem- and emotion-focused coping was higher in the intervention group immediately after intervention (*p* < 0.001) and one month after intervention (*p* < 0.001) versus controls. Lower stress in the intervention group immediately after intervention (*p* < 0.001) and even lower one month after intervention (*p* < 0.001) versus controls.	Supportive programs improve coping and mitigate stress in women with breast cancer.
Scheier (2005) [34]	Group sessions began with informational presentations followed by guided discussions of related topics. Interactions between participants were kept to a minimum. Participants were assigned to one of two intervention groups: educational or nutritional. In the educational intervention group, session topics included talking with children about cancer, managing stress and anxiety after diagnosis, relationships and intimacy, and hormones and heredity. Participants were given booklets and told of local resources for more information and support. In the nutritional intervention group, session topics included choosing healthy foods, cooking methods, and how to maintain a healthy diet while eating out. Participants also kept a food diary for four days.	Participants in the control group received standard medical care.	4 months	Coping (affect), coping (spiritual), self-monitoring of skills/thoughts/etc., self-help materials (bibliotherapy)	At 9 months post intervention, participants in the nutrition arm had lower depressive symptoms (*p* < 0.001) versus controls. At 9 months post intervention, participants in both the nutrition arm (*p* < 0.02) and the education arm (*p* < 0.001) had better physical functioning versus controls. Overall mental health functioning improved over time (*p* < 0.001); however, there was no treatment and time interaction.	Educational and nutritional interventions enhance adjustment among young women at the end of nonhormonal adjuvant therapy.
Schover (2006) [37]	Participants were given a workbook with three chapters on menopause and breast cancer, sexuality and breast cancer, and cancer and your family. Participants met with a peer counselor for three sessions each focused on the workbook chapter. Peer counselors (culturally similar breast cancer survivors) were matched with the age of participants. Participants also received a resource list of books, Web sites, hotlines, and local clinics.	Participants were assigned to the three-month wait-list control group.	3 months	Self-help materials (bibliotherapy)	From baseline to 3 months post intervention, knowledge of reproductive issues (*p* < 0.0001), emotional distress (*p* < 0.0047), and menopause symptoms (*p* < 0.0128) improved in the intervention group versus controls. Women who were sexually dysfunctional became less distressed (*p* < 0.0167) from baseline to 3 months post intervention versus controls.	An intervention that includes peer counselors from similar cultural background can have a positive effect on knowledge and symptoms in cancer survivors.
Sturm (2014) [51]	The choreographer/dance trainer led ten 60 min group-based dance classes comprised of (1) warm-up, (2) isometric muscle work, followed by sessions on healthier movement patterns, (3) emphasis on moving through space, and (4) group choreography and cool down.	Assigned final twenty participants to the control group; participants were contacted twice per week, but no group activity; after the completion of control period, control participants were offered to participate in the group activity.	5 weeks	Relaxation training, coping (physical)	Decrease in cancer-related fatigue in the intervention group versus controls (*p* = 0.001). Intervention group had better emotional (*p* = 0.03) and social functioning (*p* = 0.008) versus controls.	Incorporating dance therapy during the early stages of cancer treatment helps to manage cancer-related fatigue.
Tabrizi (2016) [44]	In unstructured supportive expressive discussion group sessions, participants discussed psychological information, e.g., fear of recurrence, stress management, coping strategies, managing physical and mental activity, setting goals, staying positive, plans for future, and medical information; encouraged participants to increase interactions and build new bonds of social support, seek sources of support, and enhance communication; learning how to cope with the feelings of isolation and reorder life priorities.	Participants in the wait-list control group received routine care, which included a brochure regarding self-care during chemotherapy. After the completion of intervention and second assessment, the control group participated in the program.	12 weeks	Coping (affect), coping (spiritual), goal setting, cancer survivor/patient testimony	Intervention group showed a reduction in loneliness (*p* < 0.001), greater hope (*p* = 0.01), improved overall QOL (*p* = 0.002), and improved social functioning (*p* = 0.024) from preintervention to follow-up at 8 weeks. Control group did not see any significant changes in loneliness, hope, QOL, or social functioning from preintervention to follow-up at 8 weeks.	Supportive expressive discussion groups improve loneliness, hope, and QOL in breast cancer survivors.
Vos (2004) [45]	In the group psychotherapy intervention group, therapists guided participants in semi-structured group discussions about various topics including the personal meaning of having breast cancer, coping with emotions, asking for social support, giving social support, and going on without going to the group. The goal was for participants to learn about how they felt about having breast cancer, its consequences, and how to express these feelings. The same topics were discussed in the social support intervention group. In this group, there was no therapist with a manual guiding the conversation. Instead, participants shared experiences of diagnosis/treatment of breast cancer and were encouraged to receive peer support and emotional encouragement. The sessions were structured where participants, at the end of each session, decided on the topic for the next week’s session. Short coffee breaks were also provided during the social support intervention. The goal of this intervention was for participants to learn to cope with problems in a practical manner.	After the completion of intervention and assessment, the control group participants were invited to join the intervention.	12 weeks	Cancer survivor/patient testimony, problem solving	Participants in both the intervention groups combined perceived more support from others not very close to them versus controls (*p* = 0.01). Participants in the social support intervention group perceived more support from others not very close to them versus participants in the group psychotherapy or controls (*p* < 0.01). Participants in the social support used more palliative coping at 3 months post-baseline than at baseline (*p* = 0.05).	Group psychotherapy and social support interventions do not have many short-term benefits on psychosocial outcomes for breast cancer patients.
Vos (2007) [46]	In the group psychotherapy intervention group, therapists guided participants in semi-structured group discussions about various topics including the personal meaning of having breast cancer, coping with emotions, asking for social support, giving social support, and going on without going to the group. The goal was for participants to learn about how they felt about having breast cancer, its consequences, and how to express these feelings. The same topics were discussed in the social support intervention group. In this group, there was no therapist with a manual guiding the conversation. Instead, participants shared experiences of diagnosis/treatment of breast cancer and were encouraged to receive peer support and emotional encouragement. The sessions were structured where participants, at the end of each session, decided on the topic for the next week session. Short coffee breaks were also provided during the social support intervention. The goal of this intervention was for participants to learn to cope with problems in a practical manner.	Therapist guided group discussion about various topics including the personal meaning of having breast cancer, coping with emotions, asking for social support, giving social support, and going on without going to the group. Participants learned about how they felt about having breast cancer, its consequences, and how to express these feelings.	12 weeks	Cancer survivor/patient testimony, problem solving	Positive changes for body image and recreation regardless of the type of intervention. For both groups combined, at 12 months post intervention, participants reported better body image (*p* = <0.001) and that their illness had less impact on recreational activities (*p* < 0.001) versus baseline.	Group psychotherapy and social support interventions may improve body image and recreation (a subscale of social adjustment) for breast cancer patients.
Zhang (2017) [39]	A psychologist led a mindfulness-based stress reduction program that provided supportive interaction among the intervention group participants. The intervention consisted of four basic forms of meditation practice (body scan, walking meditation, yoga, and sitting meditation), group discussions, didactic teaching, and home practice. Participants discussed their experiences related to the intervention.	Participants in the control group received usual care. Participants were offered to participate in the intervention if they desired after the completion of the study period.	8 weeks	Relaxation training, practice new skills in or outside the intervention	Intervention group showed significant improvement in posttraumatic growth after the 8-week intervention (Cohen’s d = 0.38, *p* < 0.001) versus controls. Intervention group had lower perceived stress (Cohen’s d = 0.21, *p* < 0.001) and anxiety (Cohen’s d = 0.21, *p* < 0.001) versus controls.	A mindfulness-based stress reduction intervention can reduce stress and anxiety in Chinese breast cancer patients.

Note: QOL = quality-of-life. In all interventions, participants were randomly assigned to intervention or control groups, except Sturm (2014) assigned the first twenty participants to the intervention group and the final twenty participants to the control group.

**Table 4:** Mixed-type (technology- and non-technology-based) interventions, social integration component of the intervention, results, and implications of the interventions.

Author (Year)	Intervention(s) Description	Control Group (CG) Description	Intervention Duration	Social Cognitive Theory Component of the Intervention	Results	Implication
Chen (2019) [53]	Participants were given an educational manual based on social interaction theory that focused on survivors’ experiences with treatment, side effects, hygiene, social interaction skills, and supportive psychological care. Then, a nurse facilitated the behavior changes and health education group sessions focused on social interactions, verbal/nonverbal behaviors used for communication and interaction with others, and group discussions. A video of the course was provided for practice after the lessons. After the course ended, telephone calls were made to remind participants about the course content and the importance of behavior modification.	Participants in the control group received routine care only and were given the option to participate in the intervention after the trial completion.	12 weeks	Coping (physical), coping (affect), practice new skills in or outside the intervention, cancer survivor testimony	Intervention group reported less fear of social interactions (*p* < 0.05), less avoidance of social interactions (*p* < 0.05), and better physical function (*p* < 0.01) versus controls. Intervention group at 3 months post intervention had less depression than at baseline (*p* < 0.001).	Behavior changes and health education intervention can improve social interactions and health outcomes in head and neck cancer survivors.
Lee (2013) [47]	Peer support partners met with participants either face-to-face or by telephone for at least 20 min. Face-to-face meetings were conducted at a coffee shop or at the patient’s home to provide a comfortable environment to chat with their peer. Intervention emphasized a supportive environment, helping to reduce emotional distress or physical discomforts, as participants discussed problems typical after surgery. The intervention provided one-on-one interaction and mutual support, sharing feelings, information, and promotion of self-confidence.	Participants in the control group received usual care only.	6 weeks	Self-monitoring of skills/thoughts/etc., cancer survivor/patient testimony	Intervention group reported an increase in self-efficacy for self-management (*p* = 0.043) versus controls No significant differences were observed in anxiety, depression, and mental adjustment between the groups.	Dyadic peer support intervention can increase self-efficacy among newly diagnosed breast cancer patients.
Park (2012) [48]	Participants were provided individual face-to-face education with a handbook with information on the survivors’ experience, six telephone-delivered health coaching sessions, and three small group meetings. The individual meetings focused on preventing, identifying, and resolving problems and developing coping and management strategies. Telephone sessions focused on individualized management plans. Group meetings included 5–8 women who discussed common health issues they experienced, emotional stress, burden, diet, and exercise.	Participants received standard care from their medical team. They received a short booklet with cancer information, treatment adverse effects, follow-up care, and healthy eating and were suggested to contact their healthcare team for follow-up care. At the end of the study, the control group participants were invited to participate in the intervention program.	12 weeks	Coping (physical), coping (affect), cancer survivor/patient testimony	Intervention group had higher QOL (*p* = 0.009), social well-being (*p* = 0.032), emotional well-being (*p* = 0.031), functional well-being (*p* = 0.036), lower overall symptoms (*p* = 0.011), and lower psychological symptom distress (*p* = 0.032) versus controls. There was also a significant time-by-group interaction effect for QOL (*p* = 0.014), emotional well-being (*p* < 0.001), overall symptoms (*p* = 0.001), and psychological symptom distress (*p* < 0.001), where the intervention group improved over time versus controls.	The psychoeducational support program may improve QOL and symptom experiences in breast cancer survivors.
Ye (2016) [40]	Peer mentors (breast cancer survivors) were matched with a participant based on demographics. Educational sessions included topics such as surgical treatment, music therapy, traditional Chinese medicine, and Taiichi. Educational sessions were followed by group discussions where mentors and mentees (usually in 2–3 pairs) discussed topics and mentors provided support and advice, shared personal feelings, and mentees could ask any questions. Small group discussions between mentor and mentee provided opportunities for participants to share any personal feelings that they would not feel comfortable sharing in a large group setting. Mentors called mentees at least once per week to remind them of the upcoming session and see if they had any concerns.	Participants in the control group received usual care. Participants were offered to participate in the intervention after the completion of the study.	8 weeks	Cancer survivor/patient testimony	At 2 months, the intervention group had lower depression (Cohen’s d = 0.65, *p* = 0.0019), better hope (Cohen’s d = 0.81, *p* < 0.001), and better QOL (Cohen’s d = 0.60, *p* = 0.002) versus controls. At 6 months, the intervention group had lower anxiety (Cohen’s d = 0.74, *p* < 0.001), fatigue (Cohen’s d = 0.65, *p* < 0.001), better social support (Cohen’s d = 0.51, *p* = 0.009), and more resilience (Cohen’s d = 0.83, *p* < 0.001) versus controls. At 12 months, the intervention group reported better cognitive function (Cohen’s d = 0.55, *p* < 0.001) versus controls.	Multidiscipline mentor-based intervention may improve positive health outcomes and reduce the risk of distress associated with breast cancer.

Note: QOL = quality-of-life. In all interventions, participants were randomly assigned to intervention or control groups, except Park (2012) randomized using the even and odd number in the last digit of the participant’s identification number.

## Data Availability

The data from this study are available from the PubMed, CINAHL, and Web of Science databases.

## Supplementary Materials

The following supporting information can be downloaded at: https://www.mdpi.com/article/10.3390/cancers15194710/s1, Supplemental Figure S1: PRISMA flow diagram for study selection; Supplemental Table S1: Literature Search Strategy; Supplemental Table S2: Cochrane Risk of Bias Assessment.
